# Construing motherhood across generations in Taiwan’s social transition: associations with depression and somatic symptoms

**DOI:** 10.3389/fpsyg.2026.1655539

**Published:** 2026-02-11

**Authors:** YuChi Lin

**Affiliations:** College of Education, Chinese Culture University, Taipei, Taiwan

**Keywords:** compressed modernity, construing, depression, generation, motherhood, neoliberalism, somatisation, Taiwan

## Abstract

This study explores how Taiwanese mothers from two generations construe motherhood and how these constructions relate to depression, somatisation, and social functioning. Drawing on Personal Construct Psychology and using the repertory grid technique as a semi-structured assessment tool, the study compares mothers born before and after 1987—a pivotal year marking the lifting of martial law and the shift toward democratic, individualised parenting norms. Quantitative findings reveal distinct generational portraits. For older mothers, the public self is central; distress is linked to the pressure to maintain consistency between public performance and private reality, and unresolved intergenerational tensions are often expressed through somatic symptoms. In contrast, younger mothers exhibit significantly higher tightness of construing and construe their child as significantly closer to their ideal self, suggesting the child is central to their ideal identity. Their distress arises primarily from discrepancies between their private self and high internal standards. Additionally, household income was significantly correlated with mental health only for younger mothers, suggesting that financial resources function as a subjective condition for coping with intensive parenting demands. The study highlights how generational shifts influence the vulnerabilities of caregiving, suggesting that policy support for the younger generation should go beyond financial subsidies to focus on bolstering social networks.

## Introduction

Scholars have described post-war Taiwan as undergoing a highly accelerated and compressed process of modernisation and democratisation, with particularly dramatic shifts occurring after martial law was lifted in 1987 (Chang, 2010; Lan, 2014). Preceding this change, Taiwan experienced rapid economic growth and expanding middle-class dissatisfaction with political restrictions. These domestic and external pressures culminated in the lifting of martial law, which dismantled bans on political association and speech. This rapid transformation not only reshaped political structures and social values but also led to pronounced generational differences, particularly in the realm of parenting ideologies and practices (Lan, 2014).

In the immediate postwar decades, parenting in Taiwan has been characterised as a predominantly disciplinary task, with parents expected to enforce obedience, reflecting broader authoritarian social norms (Lan, 2014). From the 1970s onwards, however, a noticeable shift took place. The government and experts began promoting “parental education,” encouraging parents to become learners themselves, absorbing knowledge about child development and psychological health (Lan, 2014). After democratisation in 1987, expectations for parenting shifted further toward emotional attunement and mutual respect. Parents were no longer just figures of authority; they were now expected to build relationships grounded in empathy, equality and trust. Lan (2014, 2019, 2025) argues that this change brought with it a new form of parental labour—“emotional labour”—that demanded not only time and energy but also a deep awareness of the child’s psychological and emotional world.

While 1987 not only marks Taiwan’s political democratisation, it also represents a broader turning point in cultural and educational reforms that profoundly influenced family life and parenting ideologies. In the decades following democratisation, Taiwan experienced rapid expansion of higher education for women, growing exposure to global parenting discourses and increased public promotion of psychological expertise in child development (Lan, 2019; Silverman and Chang, 2015). Under the influence of neoliberalism, childrearing further shifts from obedience-oriented parenting models toward emotionally attuned, expert-informed and highly individualised caregiving ideals.

Consequently, contemporary mothers are often tasked with contradictory mandates: they are expected to pursue self-actualisation as autonomous individuals while simultaneously engaging in “intensive mothering” (Lan, 2019). Scholars have described this as a neoliberal double bind (Vandenbeld Giles, 2014), where the responsibility for balancing these competing demands—professional success and domestic perfection—is placed entirely on the individual mother. Under this logic, any difficulty in managing these roles is often perceived not as a structural issue, but as a personal failure of self-management (Rottenberg, 2018). This theoretical framework helps explain why the “liberated” younger generation might paradoxically experience heightened anxiety and self-surveillance compared to the older generation.

In this article, generational experiences therefore do not simply refer to chronological age, but to differences in socialisation contexts before and after this transition. Mothers born before 1987 grew up largely under stronger family hierarchy, more limited educational opportunities and more traditional gendered expectations of maternal self-sacrifice, whereas mothers born after 1987 came of age in a more democratic, globalised and market-oriented Taiwan, with expanding university enrolment, rising dual-earner households and increasingly individualised parenting discourses (Chang, 2010; Lan, 2014, 2019; Yang et al., 2020). The aim of the present study is not to identify specific historical events as direct causes of individual differences, but to describe cohort-based patterns of construing that have emerged within these changing cultural conditions.

Women are the focus here because Taiwanese women have been described as particularly affected by these transformations over recent decades (Silverman and Chang, 2015). However, it is important to note that focusing on mothers does not imply that parenting is solely a maternal responsibility. Rather, it reflects the gendered reality of Taiwan’s family structure, where the emotionally distant authoritarian father often leaves the mother as the primary figure for emotional labour (Lan, 2014). By examining mothers’ construing, this study aims to understand how women navigate their identity within this specific patriarchal division of labour, rather than to pathologise the maternal role in isolation. The dramatic decrease in fertility rates after 1987 has been noted as coinciding with these shifts: more women began to choose lower levels of fertility to pursue alternatives to a life dominated by childrearing (Chen, 2012). Within this context of compressed modernity, following Chang’s (2010) description of a setting in which economic, political, social and cultural changes are compressed in time and space, women born before and after 1987 are likely to differ not only in their external parenting practices but also in the underlying construing systems they attach to the maternal role.

Women born before 1987 grew up in a relatively traditional, obedience-oriented family environment, but when they became mothers they found themselves in a cultural context that expected them to parent differently, emphasising emotional connection and shared decision-making with their children. What they carry into motherhood is therefore not a single, inherited model that must simply be unlearned. Rather, their personal construing systems are shaped by a layering of old and new values—some absorbed from their childhood environments, others encountered and negotiated throughout adolescence and early adulthood. This layered and sometimes contradictory set of expectations may create internal tensions, especially when conflicting constructions coexist within the self. In such cases, distress may arise not only from difficulty in adapting to new ideals but also from the emotional labour of holding together conflicting meanings of what is a “good mother.” Previous research has examined how Taiwanese mothers construe their “public self” (self being with the child in public) and “private self” (self being with the child in private) in relation to their child, and how the perceived distance between these two roles relates to mental well-being (Lin, 2025a). In this context, the public self refers to how a mother behaves and evaluates herself when she and her child are in social settings, under the gaze of others; the private self reflects how she experiences and expresses herself at home, in a space with fewer external social expectations. Earlier findings suggested that when these two selves are construed as highly similar—meaning mothers feel unable to relax or shift roles across contexts—depression and somatisation are more likely to occur. Conversely, mothers who can differentiate their public and private selves tend to show greater behavioral flexibility and report better mental health.

However, this study did not distinguish between younger and older generations of mothers. The pattern which has been documented—particularly the heightened concern with public evaluation—may reflect the experiences of mothers who grew up under more traditional expectations surrounding reputation, discipline and social propriety.

On the other hand, it is possible that younger mothers may be more concerned with private than public evaluation. Younger mothers are raising children in a parenting environment that has become increasingly individualised and privatised. While they may not experience the same internal tensions caused by conflicting value systems relating to other people’s gaze, some may hold firmer convictions about what parenting should involve, shaped by the discourses they encountered throughout their development.

Consequently, relatively firm and clearly articulated beliefs regarding childrearing may be more prevalent among younger mothers than older ones, creating a different type of psychological pressure. In this research, this relative firmness of construing is examined through what Kelly (1955) termed tightness, a type of construing in which the individual makes ‘unvarying predictions’. Operationally, tightness is defined as the percentage of variance explained by the first principal component in the analysis of a repertory grid, the principal technique developed by Kelly to explore the structure and content of construing. Theoretically, a large first principal component in the grid analysis indicates a “tight” or unidimensional cognitive structure, where the individual relies heavily on a single, dominant dimension (e.g., good vs. bad) to interpret a wide range of experiences (Winter, 1992). While such rigidity can serve as a defence mechanism to render the world more predictable, it has also been linked to psychological vulnerability. As Winter (1992) and Neimeyer (1985) suggest, when the self is construed entirely through a rigid, superordinate standard, any failure to meet this standard can result in a collapse of the construct system, precipitating depression or anxiety.

These more firmly structured beliefs regarding childrearing may also be linked to how mothers experience financial pressure. In earlier decades, extended family members—such as grandparents and relatives—often played an important role in daily childcare, providing informal support that helped reduce the perceived financial and emotional burden of raising children. As Taiwan has moved towards more individualised and privatised forms of parenting, informal support networks appear to have either weakened or been strategically avoided to maintain parenting autonomy. Consequently, parents increasingly describe themselves as primarily responsible, both practically and financially, for their children’s care and education (Lan, 2019, 2023). Sociological studies indicate that, reflecting a phenomenon of the monetisation of care, younger mothers in post-authoritarian Taiwan are more likely to endorse intensive, education-oriented parenting ideals and to describe a strong sense of responsibility for optimising their children’s opportunities (Lan, 2019; Hong et al., 2025).

Nevertheless, recent statistics indicate that total expenditure on children has risen across families nationwide: monthly spending has increased for both preschool-aged children (0–6) and school-aged children (6–12) (Ministry of Health and Welfare, Taiwan, 2022; Chen and Lee, 2021). These trends suggest that families of different generations are now raising children in a shared context of high childrearing costs, rather than that only one cohort faces economic strain. Beyond objective indicators of income or expenditure, several studies have highlighted the role of subjective economic strain. Rather than describing absolute poverty, younger cohorts often speak of “not having enough” in terms of perceived insecurity, difficulty maintaining an expected standard of living, or anxiety about future childrearing and educational costs (Chen, 2020; Foundation for Future Generations, Center for Sustainable Democracy, 2022).

In this sense, financial pressure functions less as a direct reflection of actual income and more as a subjective experience of one’s overall life constraints. For some younger adults, money-related worries may therefore be partly decoupled from their objective economic position. Yet it remains unclear whether such feelings of “never enough” are specific to younger mothers or are also shared by older mothers, and how different generations construe financial issues in relation to their own distress.

The aim here is not to compare objective childrearing expenditures, but to examine the subjective meanings mothers attach to financial demands. Specifically, using a Personal Construct Psychology framework, the study investigates how these economic concerns are positioned within mothers’ construing of self and relationships, and how they connect to depression and somatic symptoms.

The focus on subjective meaning is critical because specific characteristics of construing have been shown to relate to mental health conditions. As demonstrated in prior studies (Lin and Winter, 2022; Lin, 2025a), rigid construing patterns are closely associated with psychological distress in Taiwanese women. As noted earlier, such tight construing has been linked to somatic symptoms, suggesting that somatisation may emerge when the interpretative system becomes too rigid to accommodate new experiences (Lin, 2025a). Furthermore, the transition to motherhood often intensifies these vulnerabilities. Past literature indicates that mothers report higher levels of depression and somatic symptoms than the general population, which are frequently linked to reactivated attachment patterns and early family experiences (Lin and Payne, 2014).

Such an inquiry is grounded in the understanding that construing is not merely a cognitive process but an embodied one. Consistent with this view, depression has been associated with greater perceived dissimilarity between the self and social roles, as well as with internal dilemmas involving how others want them to be—particularly in maternal and familial contexts (Lin and Winter, 2022). These findings highlight that suffering manifests in both body and mind and is not simply the result of individual difficulties, but reflects tensions embedded in self-construing and cultural expectations.

Importantly, however, these earlier studies did not explicitly incorporate a generational framework. While they revealed meaningful links between construing and symptoms, they did not examine whether these patterns vary across cohorts shaped by different social and historical conditions. It remains an open question whether women of different generations construct motherhood—and experience distress—in similar ways, or whether the meanings and implications of certain construing patterns differ depending on one’s generational context. Addressing this question requires closer examination of how generationally situated meaning systems relate to specific forms of psychological distress.

Despite increasing attention to structural and cultural shifts across generations, integrating these perspectives into the understanding of maternal well-being remains a critical task. While sociological scholarship has explored transformations in family norms and intergenerational expectations, addressing maternal distress solely through individual or clinical frameworks risks overlooking the broader social and temporal contexts in which distress is situated. Such a narrow focus leaves the subjective psychological experiences of mothers in Taiwan’s changing cultural context underexplored. Yet, growing evidence suggests that how mothers interpret and respond to these social ideals is deeply embedded in generational narratives and historical conditions, shaping both their emotional and embodied experiences.

In light of these concerns, this study uses the repertory grid technique, grounded in Personal Construct Psychology (PCP), to explore how Taiwanese mothers from different generations construe motherhood. The repertory grid allows both individual and generational patterns of meaning-making to emerge, offering insight into how mothers organise and differentiate key constructs related to maternal roles and parenting. By examining these meaning systems in relation to depression and somatic symptoms, the study aims to clarify how psychological distress is shaped not only by internal dynamics but also by the cultural and historical conditions in which women become mothers.

### Hypotheses

The aim of this research is to investigate generational differences in how Taiwanese mothers experiencing varying levels of depressive and somatic symptoms construe childrearing and their experience of motherhood. The repertory grid technique (RGT), a common tool to explore individuals’ construal, and questionnaires were adopted. This paper draws upon data from a study of how Taiwanese mothers with depression and somatisation construe themselves in the public and private sphere while being with their child (Lin, 2025a) to examine whether there are differences of construing about childrearing between generations who were born before and after 1987.

The hypotheses were outlined as follows:

1. household income is more crucial to younger mothers’ mental health than to older mothers
2. younger mothers have higher level of tightness regarding childrearing than older mothers
3. for older mothers, the public self will be more central in their construing, reflected in smaller distances between this element and other core elements such as the private self
4. for younger mothers, the private self will be more central in their construing, reflected in smaller distances between this element and personally salient elements such as the ideal self and the self before having a child

## Methods

### Participants

Participants were self-identified mothers who were members of private Facebook groups for mothers in Taiwan. Women were eligible to take part if they (a) reported experiencing feelings of depression related to childrearing, (b) felt pressure regarding childrearing, or (c) had a general interest in exploring mothers’ inner world in the context of childrearing. The author did not require participants to exceed a specific cut-off score on the depression measure; instead, the study was designed to include mothers with varying levels of depressive symptoms.

63 Taiwanese mothers participated in this study, with an average age of 36.08 years (S.D. = 4.58). 36 participants were in the older mothers group, while 27 participants were in the younger mothers group. The average age of the older mothers group was 39.17 years (SD = 2.84; range = 36–49), whereas the average age of the younger mothers group was 31.96 years (SD = 2.89; range = 22–35). This research (project no. 108-WFD-07-10447) was reviewed and approved by the Research Ethics Committee of National Taiwan Normal University in accordance with relevant ethical standards.

### Procedure

This study was conducted in Taipei, Taiwan in 2020. After reading the consent form, participants first completed a demographic questionnaire, followed by two self-rated questionnaires and a repertory grid interview. Participants were recruited through Facebook advertisements posted in private groups for mothers in Taiwan. The advertisement invited mothers who were experiencing depression related to childrearing, feeling societal or peer pressure, or who had a general interest in the inner experiences of motherhood. Interested mothers contacted the author directly for further information and participation.

### Measures

#### Edinburgh Postnatal Depression Scale (EPDS)

The Edinburgh Postnatal Depression Scale (EPDS; Cox et al., 1996) is widely used to screen for postnatal depression and has been frequently applied in Taiwan. It contains 10 items, each scored from 0 (not at all) to 3 (as much as I always could). A total score above 13 suggests a high likelihood of depressive symptoms.

#### The Screening for Somatoform Symptoms-7 (SOMS-7)

Rief and Hiller (2003) developed the SOMS-7 based on DSM-IV and ICD-10 criteria. The scale includes 53 items, each rated from 0 (not at all) to 4 (very severe), and participants are asked to report symptoms for which doctors could not find a clear medical cause. In the present study, the author calculated a severity index by summing all item scores, with higher scores indicating a greater number and/or severity of somatic symptoms. The SOMS-7 was originally designed as a dimensional measure to detect changes in symptom severity and frequency rather than to provide a single diagnostic cut-off. Accordingly, SOMS-7 scores in this study were treated as a continuous measure of somatisation. In 2015, a Taiwanese psychiatrist translated the scale into Chinese and examined its accuracy (Lin, 2016).

#### Social Functioning Scale (SFS)

SFS was designed to assess the social functioning of individuals with psychiatric conditions in community settings (Song, 2001). The scale consists of 36 items and covers seven subdomains: social engagement/withdrawal, interpersonal communication, independence–competence, independence–performance, recreational activities, prosocial behavior, and employment/occupation. Each item is rated on a four-point scale, with total scores ranging from 1 to 100; higher scores indicate better social functioning. Respondents are instructed to evaluate the individual’s general living situation over the past three months. The SFS has been widely used in medical and rehabilitation institutions across Taiwan and has demonstrated good reliability and validity.

#### Repertory grid technique

The repertory grid technique (RGT), originally introduced by Kelly (1955), is a widely used method in personal construct psychology (PCP) to explore how individuals interpret their experiences (Hardison and Neimeyer, 2012). Within PCP, individuals are viewed as “scientists” who anticipate events through their own system of bipolar constructs—such as happy vs. sad—each with a range of convenience (Fransella et al., 2004). The RGT helps examine how people understand a particular topic through four core components: topic, elements, constructs and ratings. It is administered as a semi-structured interview, in which the interviewer guides participants to articulate their own meanings.

In the present study, the RGT was used to capture mothers’ idiosyncratic constructs regarding caregiving. The grid included 12 elements: actual self, ideal self, ideal mother, my mother, my child, ideal child, self with child in private, self with child in public, self as a child, self before becoming a mother, husband and mother-in-law. Following Jankowicz’s (2004) suggestion, ten constructs were elicited. All 12 elements were presented to each participant in groups of three. In each triad, participants were asked: “Could you please find an important way in which two of these are alike and thereby different from the third?” This elicited the emergent pole of a construct. Participants were then invited to generate the implicit pole. This procedure was repeated until 10 constructs were elicited. Finally, participants rated every element on each construct using a 7-point scale.

Regarding the variables used in the present study, two key measures were derived from the grids using the single-grid Slater analysis (Grice, 2002; Slater, 1977). The first measure was tightness (percentage of variance explained by the first principal component), which reflects the degree of cognitive structure. A higher percentage suggests that the participant’s constructs are more tightly organised. The second measure was the psychological distance between selected elements. Greater values represent stronger perceived differences between those elements in the participant’s personal construct system.

#### Data analysis

Descriptive statistics (means, standard deviations, frequencies and percentages) were first computed for all demographic and clinical variables in the total sample and separately for the two generational groups. Group differences in categorical variables (occupation, education, marital status, number of children and household monthly income) were examined using chi-square tests. For continuous variables (e.g., mothers’ age, children’s age, depression, somatisation, social functioning, tightness and distances between elements), between-group comparisons were conducted using independent-samples t-tests; where assumptions of normality or homogeneity of variance were clearly violated, parallel Mann–Whitney U tests were calculated as a robustness check. In the demographic questionnaire, ‘household monthly income’ was defined as the total combined monthly income of the nuclear family (typically comprising the participant, her spouse/partner, and children). This measure was adopted to assess the overall financial resources available to the household for sustaining the family unit and coping with childrearing demands, rather than focusing on the mother’s individual earnings.

To address Hypothesis 1, the associations between household monthly income and depression, somatisation and social functioning were examined separately for older and younger mothers using Pearson correlation coefficients. To address Hypothesis 2, tightness was operationalised as the percentage of variance explained by the first principal component in the single-grid Slater analysis and compared between generational groups using independent-samples t-tests. To address Hypotheses 3 and 4, psychological distances between selected elements in the grids were obtained from the same Slater analyses. Group differences in these distances were examined using independent-samples t-tests. Within each generational group, Pearson correlations were then calculated between tightness, element distances and indicators of mental health (depression, somatisation and social functioning) to identify construing patterns associated with mental health.

All analyses were conducted using IBM SPSS Statistics (version 28). A two-tailed alpha level of 0.05 was used for all statistical tests.

## Results

### Comparison between the older mothers group and the younger mothers group

#### Demographic information

There were no statistically significant differences between the older and younger mothers in occupation, education, household monthly income, marital status or number of children (Table 1). By contrast, the age of children in the older mothers group was significantly higher than that in the younger mothers group (*p* < 0.013), which is consistent with the expectation that younger mothers are more likely to have younger children.

**Table 1:** Demographic Characteristics and Comparisons Between Older and Younger Mothers.

	Older mothers group Age > 35 (*n* = 36)	Younger mothers group Age ≤ 35 (*n* = 27)	All (*n* = 63)	*P*
Age	39.17 ± 2.84	31.96 ± 2.89	36.08 ± 4.58	<0.001
Employment status				0.658
Housewife	14 (38.89%)	12 (44.44%)	26 (41.27%)	
Full−/part time Employed	22 (61.11%)	15 (55.56%)	37 (58.73%)	
Education				0.231
Undergraduate	20 (55.56%)	19 (70.37%)	39 (61.90%)	
Graduate	16 (44.44%)	8 (29.63%)	24 (38.10%)	
Household monthly income				1.000
0–100,000 (N.T.)	24 (66.67%)	18 (66.67%)	42 (66.67%)	
>100,000 (N.T.)	12 (33.33%)	9 (33.33%)	21 (33.33%)	
Marriage				0.140
Unmarried	2 (5.56%)	0 (0.00%)	2 (3.17%)	
Married	34 (94.44%)	26 (96.30%)	60 (95.24%)	
Separated	0 (0.00%)	1 (3.70%)	1 (1.59%)	
Children’s age				0.017
Under 2-year-old	8 (22.22%)	12 (44.44%)	20 (31.75%)	
2–7	23 (63.89%)	15 (55.56%)	38 (60.32%)	
Over 7	5 (13.89%)	0 (0.00%)	5 (7.94%)	
Children’s average age (month)	43.56 ± 34.64	24.89 ± 17.04	35.56 ± 29.76	0.013
Number of children	1.53 ± 0.65	1.33 ± 0.55	1.44 ± 0.62	0.218

#### Comparison of depression, somatisation, social function, and household income

The self-rated Edinburgh Postnatal Depression Scale (EPDS) scores indicated that, in the older mothers group, 12 participants were below the recommended cutoff score for probable depression and 24 were above it. In the younger mothers group, 6 participants were below the cutoff, whereas 21 were above it. As summarised in Table 2, mean depression and somatisation scores did not differ significantly between generations. However, younger mothers showed a tendency toward higher levels of both depressive symptoms and somatic complaints compared with older mothers, even though these differences did not reach statistical significance (Table 2).

**Table 2:** Comparison of Mental Health-Related Variables and Tightness of Construing Between Older and Younger Mothers.

	Older mothers group	Younger mothers group	All (*n* = 63)	*P*
Depression	12.44 ± 4.98	13.85 ± 4.97	13.05 ± 4.99	0.271
Somatisation	39.33 ± 23.63	42.15 ± 20.92	40.54 ± 22.38	0.625
Social function	99.33 ± 12.52	91.85 ± 15.25	96.13 ± 14.14	0.037
Tightness	53.42 ± 10.04	58.97 ± 11.93	55.80 ± 11.15	0.0498

The older mothers group had significantly higher social functioning than the younger mothers group (Table 2). Moreover, in the younger mothers group, higher household monthly income (coded as ≤100,000 vs. >100,000 N. T. dollars per month) was significantly negatively correlated with both depression and somatisation, and positively correlated with social functioning, whereas no such correlations were found in the older mothers group (Table 3). In addition, within the younger mothers group, poorer social functioning was significantly associated with higher depression scores (r = −0.52, *p* = 0.006; analysis not shown in tables). There were no significant group differences in depression or somatisation by children’s age; however, social functioning differed significantly across age groups, suggesting that mothers of older children tended to report better social functioning (Table 4).

**Table 3:** Correlations between household income and mental health-related variables in both groups.

Household income	Depression (*p*)	Somatisation (*p*)	Social function (*p*)	Tightness (*p*)
Younger mothers group	r = −0.41 *p* = 0.032*	r = −0.49 *p* = 0.009**	r = 0.39 *p* = 0.047*	r = −0.027 *p* = 0.172
Older mothers group	r = −0.010 *p* = 0.562	r = 0.02 *p* = 0.907	r = 0.13 *p* = 0.437	r = 0.05 *p* = 0.796

*p < 0.05; *p < 0.01.

**Table 4:** ANOVA results for child age group differences in maternal psychological outcomes.

Mental health-related variable	Younger mothers Mean (SD)	Older mothers Mean (SD)	*F*-value	*p*-value
Depression	14.60(5.53)	12.45(4.43)	1.54	0.222
Somatization	42.15(25.78)	39.26(21.73)	0.16	0.851
Social functioning	89.20(12.63)	98.08(14.02)	5.54	0.006**
Tightness	57.32(12.21)	55.02(10.54)	0.27	0.762

Child age groups were categorized as: ≤2 years, 2–7 years, and ≥7 years. **p < 0.01.

Given the small number of participants with children over 7 years of age and the uneven distribution across child-age categories, the author did not conduct additional *post hoc* comparisons by child-age group.

#### Comparison of tightness and distances between elements in two groups

The younger mothers group had statistically significantly higher tightness than that in the older mothers group (Table 2). In terms of the construing, although a range of element distances was examined, most mean distances were broadly similar across the two groups, with only the ideal self–my child distance reaching statistical significance: older mothers construed their ideal self as more distant from their child (0.98 ± 0.27) than younger mothers did (0.83 ± 0.29) (*p* = 0.041). For all other element pairs, no statistically significant differences in mean distances were found between the two groups.

#### Correlations between distances between elements and psychological distress variables in the older mothers group

The correlations between distances between elements and depression, somatisation, social function and tightness in the older mothers group indicated the significance of construing of the public self in relation to older mothers’ mental and physical health’ (Table 5). First, distance between public self and ideal mother is negatively correlated to depression and somatisation. This means that a greater discrepancy between a mother’s public self-perception and her ideal mother concept is associated with lower levels of depression and somatisation for older mothers. Second, distance between public self and my child is negatively correlated to depression and somatisation. Third, distance between public self and private self is negatively correlated to depression, somatisation and tightness.

**Table 5:** Correlations between element distances and mental health-related variables in older mothers group.

Element distances	Depression (r)	Somatization (r)	Social function (r)	Tightness (r)
Ideal mother—self with children in public	−0.429**	−0.457**	−0.025	−0.064
Ideal mother—mother	0.341*	0.340*	0.204	0.284
Self before becoming a mother—mother	0.422*	0.195	0.144	0.004
Self with children in public—child	−0.449**	−0.395*	0.260	−0.120
Self with children in public—self with children in private	−0.506**	−0.437**	0.212	−0.462**
Child—husband	−0.367*	−0.448**	0.177	0.235
Ideal child—mother	0.347*	0.416*	0.226	0.461**

*p < 0.05; **p < 0.01. Only element dyads whose distances showed at least one significant correlation (*p* < 0.05) with the mental health–related variables are reported in this table. Non-significant associations are omitted for brevity.

Finally, the distance between child and husband is negatively correlated to depression and somatisation, and distance between ideal child and mother is positively correlated to depression, somatisation and tightness.

#### Correlations between distances between elements and variables in the younger mothers group

No distances between elements involving the public self were significantly correlated with depression or somatisation. Only two distances—between ideal self and ideal mother, and between private self (self with child in private) and mother—were significantly associated with both depression and somatisation (Table 6). Greater distance between ideal self and ideal mother was related to lower levels of depression and somatisation, whereas greater distance between private self and mother was related to higher levels of both symptoms. Private self was also involved in several other associations with depression: larger distances between ideal self and private self, self before having a child and private self, ideal mother and private self, and private self and husband were all associated with higher depression scores.

**Table 6:** Correlations between element distances and mental health-related variables in younger mothers group.

Element distances	Depression (r)	Somatisation (r)	Social function (r)	Tightness (r)
Actual self—self as a child	0.436*	0.252	−0.631**	0.216
Actual self—mother-in-law	0.123	0.484*	−0.083	0.519**
Ideal self—ideal mother	−0.427*	−0.386*	0.303	−0.115
Ideal self—private self	0.411*	−0.011	−0.286	0.135
Ideal self—ideal child	−0.383*	−0.368	0.453*	−0.318
Self before becoming a mother—self as a child	−0.470*	−0.011	0.198	0.069
Self before becoming a mother—child	−0.384*	−0.203	0.307	−0.125
Self before becoming a mother—private self	0.454*	0.010	−0.226	0.041
Ideal mother—private self	0.423*	−0.011	−0.219	0.070
Self as a child–child	−0.016	0.001	0.088	−0.526**
Child—mother-in-law	−0.426*	−0.213	0.157	−0.020
Private self—mother	0.464*	0.464*	−0.342	0.207
Private self—husband	0.439*	−0.022	−0.171	0.100
Ideal child—mom-in-law	−0.495**	−0.248	0.070	−0.166
Husband—mom-in-law	−0.139	−0.301	0.153	−0.514**

*p < 0.05; **p < 0.01. Only element dyads whose distances showed at least one significant correlation (*p* < 0.05) with the mental health–related variables are reported in this table. Non-significant associations are omitted for brevity.

In addition, the distance between actual self and mother-in-law was positively related to somatisation and tightness, while greater perceived similarity between the child (both actual and ideal) and the mother-in-law was associated with higher depression scores.

## Discussion

### Demographic information

With the exception of children’s age, no significant differences were found between the two groups regarding demographic variables, depression, or somatisation. This suggests that factors beyond socioeconomic status contribute to maternal well-being, and that mothers’ construing of childrearing and self-identities may play a more central role in explaining generational differences. However, mothers of younger children reported lower social functioning, likely reflecting the practical constraints of early childcare. Notably, lower social functioning was significantly correlated with higher depression only in the younger mothers group (r = −0.52, *p* = 0.006). This suggests that younger mothers’ distress is more likely to be accompanied by observable reductions in social roles, whereas older mothers may maintain outward functioning despite experiencing considerable distress. This distinction will be further explored below in the context of neoliberal influences.

### Hypothesis 1, household income is more crucial to younger mothers’ mental health than older mothers

As hypothesised, while household monthly income did not differ significantly between generations, it appeared to play a more important role in the mental health of younger mothers. Specifically, lower household monthly income was significantly correlated with higher depression and somatisation, and lower social functioning in younger mothers, whereas these associations were weak or absent among older mothers. There are two primary interpretations for this generational discrepancy.

First, younger mothers may depend more heavily on financial resources to construct a childrearing support system that aligns with their need for autonomy. As noted in the Introduction, younger mothers tend to have firmer boundaries regarding parenting styles and are often less willing to navigate the relational obligations and potential interference that come with traditional family-based support (Shih and Pyke, 2016). Instead of relying on unpaid help from in-laws or parents—which was common for the older cohort—younger mothers may prefer to “buy” their independence through paid services, such as childcare, educational programmes, and parenting consultations (Hong et al., 2025; Lan, 2014). Consequently, for younger mothers, money is not just a material asset but the essential currency for maintaining control over their parenting environment and reducing interpersonal friction.

Second, the stronger link between income and mental health in younger mothers may be interpreted through the lens of neoliberal individualism, specifically regarding the tension between maternal devotion and self-preservation. Sociological studies suggest that unlike the older generation, who were often socialised into a script of endurance and collective family priority (Chang, 2010; Lan, 2014), the younger generation grew up in an era emphasising self-actualisation and individual competitiveness (Chang, 2010; Lan, 2019). This difference is clearly reflected in the social functioning results. Older mothers reported significantly higher social functioning regardless of their income or distress levels, suggesting that they adhere to a traditional ethic where fulfilling social roles is mandatory, even when one is suffering physically or emotionally. In other words, older mothers continue to perform their duties, which are linked to traditional female socialisation, despite personal hardship.

In contrast, younger mothers, influenced by the values of expressive individualism, may be less inclined—or feel less obligated—to suppress their discomfort for the sake of role performance. For them, financial resources are required not only for “intensive mothering” but also for maintaining an autonomous “modern self” (e.g., personal development, lifestyle, and leisure) separate from the child (Lan, 2019). When income is insufficient, younger mothers face a double bind: they cannot afford to outsource care, nor can they afford the consumption required to sustain their personal identity. Unlike older mothers who endure and keep functioning, younger mothers may experience this lack of resources as a direct threat to their self-hood, leading to a collapse in both mental well-being and social functioning.

At the same time, the present study did not assess actual childcare or educational expenditures, nor did it measure informal support from extended family members. The author therefore cannot determine whether younger mothers with higher income also invest more heavily in their children’s early education, or receive less unpaid help, in ways that might increase their sense of strain.

### Hypothesis 2, younger mothers have higher level of tightness regarding childrearing than older mothers

As hypothesised, the results indicate that younger mothers show significantly higher tightness in their construing compared to older mothers, suggesting that younger mothers tend to hold more rigid and narrowly organised beliefs about childrearing and self-identity. Building on the previous discussion of neoliberalism, this generational difference may reflect the distinct social environments in which these mothers parent. Younger mothers, navigating a competitive and risk-averse neoliberal society, may have developed strict, unambiguous standards for parenting to ensure their children’s success and money-related worries may therefore be partly decoupled from their objective economic situation to secure their own maternal identity. In contrast, older mothers may hold a looser, more flexible system of beliefs (lower tightness), possibly because they parented during a period of social transition where traditional norms and modern ideas coexisted, requiring them to adapt to diverse expectations.

Although previous studies have suggested that tightness is often linked to higher levels of somatisation and depression (Lin and Winter, 2022; Lin, 2025a), this study did not find a statistically significant difference in depression and somatisation scores between the two generational groups. One explanation is simply methodological: the higher mean scores for distress observed in the younger group (Table 2) might have reached statistical significance with a larger sample size, suggesting the possibility of a Type II error due to limited statistical power.

However, beyond statistical limitations, it is also possible that tightness does not inevitably lead to psychological distress for younger mothers because certain protective mechanisms are at play. First, value congruence with current cultural norms may buffer the negative effects of rigidity (Markus and Kitayama, 1991). Contemporary parenting culture emphasises intensive mothering; therefore, younger mothers’ rigid beliefs actually align with prevailing social ideals. Because their tight construing is validated by societal standards, they may feel a sense of correctness or moral achievement rather than conflict, thereby mitigating potential distress.

Second, perceived control over resources—as discussed in Hypothesis 1—may serve as a crucial moderator. While rigid beliefs can induce stress, it is plausible that for younger mothers who perceive they have sufficient resources to support these high standards, such rigidity might be experienced differently. If financial means allow them to enact their ideals—perhaps through accessing support or services—they may preserve a sense of parental self-efficacy (Bandura, 1997).

### Hypothesis 3, for older mothers, the public self will be more central in their construing, reflected in smaller distances between this element and other core elements such as the private self

Hypothesis 3 was partly supported for the older mothers’ group. The findings for older mothers in this study extend and refine the patterns previously identified using the same dataset (Lin, 2025a). In earlier analyses without a generational distinction, it was found that when mothers’ public and private selves became highly similar, higher levels of depression and somatisation were observed. The current generational analysis clarifies that this pattern is specific to older mothers. For this group, smaller distances between the public self and private self were significantly associated with higher levels of depression, somatisation, and tightness (Table 5). This suggests that greater consistency between the public and private self does not necessarily reflect psychological integration or well-being. Instead, it indicates a form of compulsory consistency, where mothers feel compelled to embody the rigid, idealised “good mother” image in both their private experiences and social presentations. This leaves little room for rest, ambivalence, or the expression of a personal self outside the caregiving role, thereby creating significant emotional strain (Lan, 2019; Yang et al., 2020).

Furthermore, the data show that smaller distances between the public self and the ideal mother are also associated with higher levels of depression and somatisation in older mothers. This echoes the interpretation that the emotional burden comes from the pressure to perform as the “ideal mother” in public, which prevents these mothers from relaxing or feeling authentic (Tang, 2004a). The closer their public self aligns with these internalised, high-standard ideals, the more exhausting it becomes to sustain this performance, especially in highly visible caregiving situations.

Moreover, a striking finding is that when older mothers perceive their child as resembling their public self, both depression and somatisation increase. Rather than feeling pride in raising a child who reflects a socially desirable image, these mothers appear to experience discomfort. This may be because the child is perceived as mirroring the mother’s public performance—an image that feels externally imposed rather than authentic. In this sense, the public self–child similarity crystallises a core gendered tension: older mothers are caught between the duty to transmit the “good mother” ideal to the next generation and the feeling that this ideal colonises their relationship with their child, leaving no space for their own emotional needs. This represents a central struggle for many older mothers in Taiwan: the ongoing tension between meeting external expectations and the existential question, “Can I be myself?” (Lin, 2025b).

Although these interpretations are drawn from quantitative correlations, they are supported by emerging findings from ongoing qualitative research conducted as part of a follow-up study (Lin, 2025b). In subsequent interviews, many older mothers described the struggle with authenticity—whether they are allowed to maintain a true self within the maternal role—as a central emotional issue. For them, the challenge lies in negotiating the internal conflict between personal authenticity and the socially idealised image of motherhood that they feel forced to inhabit publicly.

In contrast, preliminary qualitative data from younger mothers suggest that their emotional struggles centre more on loneliness, interpersonal distance, and the difficulty of sustaining clear personal boundaries—issues distinct from the “public mask” pressure faced by the older cohort. Taken together, these patterns suggest that older mothers’ distress is tied to the over-integration of the public role into the private self, whereas younger mothers may struggle to reconcile the contemporary ideal of self-actualisation with the intensive demands of motherhood.

### Hypothesis 4, for younger mothers, the private self with the child will be more central in their construing, reflected in smaller distances between this element and personally salient elements such as the ideal self or the self before having a child

Hypothesis 4 was supported for the younger mothers’ group. Expanding on the generational contrast noted in Hypothesis 3, the results confirm that younger mothers’ vulnerability is not linked to the public self, but is deeply centred on the private self and its relationship with internalised ideals.

According to Table 6, for younger mothers, larger distances between the private self and the ideal self, the ideal mother, and the self before having a child are associated with higher levels of depression. This provides empirical evidence that their distress arises primarily from the perceived gap between their current lived experience in private and their standards or past identity. It is understandable that distress arises when their self being with the child in private is distant from their ideal mother and ideal self, which means that the way they enact the maternal role, mother, does not meet their expectation.

Moreover, to meet the demands of caregiving—especially when they hold more rigid ideas about what good parenting should look like, and what kind of self they ought to embody in childrearing—these mothers must continually negotiate between different parts of themselves: the part that wants to be a good mother, the part that longs for personal growth, and the part that values independence or professional goals. Life after having children inevitably looks different from before, and this change is not only practical but also deeply personal. Over time, the gap between who they used to be and who they now feel expected to be may generate a subtle yet persistent sense of tension.

This tension is further contextualised by the closeness between the ideal self and the child. The group comparison revealed that younger mothers construed their child as significantly closer to their ideal self than older mothers did. This difference likely reflects the demographic reality that younger mothers have significantly younger children (average 2 years old) compared to older mothers (average 3.6 years old). Mothers of toddlers are naturally in a stage of heightened maternal preoccupation (Winnicott, 1956). However, when viewed alongside the significantly higher tightness observed in younger mothers, this closeness may also imply a more rigid construal where the mother projects her ideal self onto her child. This likely reflects the pressures of neoliberal intensive mothering, where mothers invest heavily in child-rearing and expect their child to embody their own envisioned perfection.

Meanwhile, the finding that similarity between ideal self and ideal mother is significantly associated with higher depression and somatisation further illustrates this tension. While younger mothers want to be good mothers in practice, they may feel psychologically threatened if their ideal self becomes entirely identical to the maternal role. This threat even manifests through the bodily aspect. When self-actualisation is an internalised value, younger mothers may feel that if their ideal self is completely consumed by the ideal mother—leaving no space for personal aspirations or individual desires—they have lost themselves. Thus, younger mothers may navigate a difficult psychological line: they suffer if their private performance is not good enough, but they also suffer if their ideal identity is too narrowly defined by motherhood.

This specific anxiety reflects the contradictory mandates of neoliberalism, where mothers face a double bind: they are expected to be self-optimising economic agents in the public realm, while simultaneously functioning as maternalist self-sacrificing mothers in the private realm (Vandenbeld Giles, 2014). For younger mothers, balancing these two conflicting roles is framed as a personal responsibility requiring effective self-management (Rottenberg, 2018). Consequently, any inability to perfectly harmonise these demands is perceived not as a structural issue, but as a personal failure. This pressure subjects women to a constant mode of self-surveillance in pursuit of “the perfect” (McRobbie, 2015). The high tightness found in this study may thus be understood as a manifestation of this self-monitoring, where mothers rigidly scrutinise their performance to avoid falling short in either domain.

### Mother-in-law

Finally, the study revealed notable generational differences regarding the mother-in-law. While this relationship has historically been a source of distress in Taiwan (Kung, 1999; Shih and Pyke, 2016; Sun and Lin, 2015), this study found no significant correlation between element distances involving the mother-in-law and mental health for older mothers. This lack of correlation likely does not imply that the mother-in-law is irrelevant to their well-being. Instead, it may reflect Tang’s (2004b) observation on the cultural construction of public and private spheres in Taiwan. Tang argues that unlike the Western emphasis on distinct individual boundaries, the Taiwanese “private” sphere has historically included extended kin. For older mothers, the mother-in-law may still be construed as part of this collective kinship structure rather than as an external figure to be individually negotiated with. Consequently, the boundary between self and in-law remains permeable, potentially buffering direct identity conflict.

In contrast, younger mothers—who place greater emphasis on autonomy—showed a more complex, seemingly paradoxical pattern. First, when they perceived their child (actual or ideal) as resembling the mother-in-law, depressive symptoms increased, perhaps suggesting a fear that their own parenting values were being challenged or diluted. However, this does not imply a simple desire for separation; greater distance between the actual self and the mother-in-law was associated with somatic symptoms and tightness. This indicates that while they resist assimilation, the effort to maintain a rigid distinction also generates implicit, embodied strain.

In short, unlike older mothers who may integrate the mother-in-law into their role structure, younger mothers seem to be caught between the stress of assimilation and the strain of differentiation. This generational divergence may also be explained by a restructuring of the family system associated with the life cycle stage of younger mothers, who are more likely to be actively negotiating boundaries with the extended family. As current literature provides limited insight into these dynamics, this ongoing negotiation between family obligation and parenting autonomy merits further investigation.

### Intergenerational representations of mothers in self-construing

Construing relating to the role of one’s own mother appears to contribute to psychological vulnerability across generations, reflecting the continuing influence of early caregiving experiences on psychological outcomes.

For older mothers, greater distances between the ideal mother and mother, as well as between the ideal child and mother, are associated with higher levels of depression and somatisation. This suggests that they may have experienced an unsatisfying or emotionally distant childhood and do not fully recognise or accept the parenting they received. As a result, their ideal images of what a mother or a child should be are construed as distant from their own mother. This pattern is further reflected in the finding that a greater distance between the mother and the self before having a child is also related to depressive symptoms. This echoes the possibility that emotional disconnection from their own mothers may have remained unresolved and resurfaced during their own mothering process.

Previous studies have shown that somatisation is often linked to early traumatic experiences (Alexander, 1934; Bronstein, 2011; Lin and Payne, 2014). In the context of older mothers in Taiwan, it is important to consider that such trauma may not be purely interpersonal, but also sociocultural in nature. Many of them grew up during a period of rapid societal change, where parenting practices often involved physical punishment, authoritarian discipline, or limited attention to the child’s inner world. These experiences, though once considered normative, may have become internalised as early psychological wounds.

As these women later encountered new childrearing ideas—often emphasising empathy, autonomy, and emotional attunement—the gap between how they were raised and how they now believe a child should be raised could deepen. If they do not identify with their own mother’s approach, becoming a mother themselves may intensify the emotional divide across generations. This unresolved tension, rooted in both personal history and broader sociocultural shifts, may contribute to their heightened vulnerability to depressive and somatic symptoms.

Among younger mothers, vulnerability appears to be more closely tied to everyday caregiving experiences. In this context, greater distance between the private self and the mother is associated with both depression and somatisation, suggesting that psychological strain may emerge when younger mothers perceive their way of being with their child as different from how they remember their own mother.

However, this perceived difference does not necessarily indicate disapproval or rejection. Unlike older mothers, whose distancing often involves conscious ideological disagreement, the gap observed in younger mothers may be more situational and less evaluative. It may reflect a subtle, embodied awareness that “I do it differently” in the lived moment of caregiving. Importantly, this sense of difference can carry various meanings: it could stem from feeling inadequate compared to one’s mother, or from feeling proud of doing things differently—either way, it underscores a dissonance that becomes psychologically significant.

What distinguishes this from the pattern in older mothers is that the tension here does not reside primarily in ideals, but in the intimate, daily experience of being a mother. While becoming a mother can trigger unresolved emotions or memories related to one’s own upbringing (Breen, 1975; Klainin and Arthur, 2009; Yang et al., 2005), it remains unclear why such a difference in self-perception leads to depression and somatisation. Further research is therefore needed to clarify whether this reflects an inner conflict about maternal identity, a deeper unease about inhabiting a caregiving role that feels unfamiliar or fragmented, or other contextual factors not captured in the present study.

While this study reveals significant links between the construing of the mother and participants’ distress, it is crucial to critically reflect on the absence of the participants’ own fathers in the analysis. The decision to focus on the maternal lineage was driven by the aim to explore intergenerational continuity in caregiving roles. However, this exclusion runs the risk of inadvertently reinforcing a “mother-blaming” narrative, where the origins of distress are traced solely to maternal deficiencies while the father’s role remains unexamined.

In the context of Taiwan’s patriarchal history, the father’s influence has often been characterised by authority or emotional distance rather than intimate interaction. This absence is not accidental but structural, reflecting a gendered division of labour that has historically exempted men from the emotional responsibilities of caregiving (Lan, 2014). Consequently, the distress observed in this study in relation to the participants’ mothers may not necessarily stem solely from the mother’s behavior. Instead, it is plausible that such distress is intensified because the mother was often the sole available figure for emotional connection within a family system where the father played a peripheral role.

By omitting the father element, this study captures how women navigate the maternal bond but leaves the impact of paternal absence in the background. Future research must explicitly incorporate the father figure to avoid pathologising the mother-daughter relationship and to fully understand how the patriarchal division of emotional labour shapes the intergenerational transmission of trauma.

### The role of the spouse in generational construing

While this study focuses primarily on mothers, the construing of the husband reveals a critical dimension of the relational context in which these women parent. The results indicate that the spousal relationship holds different psychological significance for the two generations.

For older mothers, the findings highlighted a distinct pattern regarding the husband and the child. As noted in the results, a greater distance between the husband and the child was associated with lower depression and somatisation. This implies that when the husband is construed as distinct from the child, he is perceived as a functional adult figure. Conversely, when the husband is perceived as too similar to the child, he may be experienced as “another child” in the family system—potentially reflecting a traditional dynamic where the husband, like the child, functions primarily as a recipient of care rather than a provider of it. For this generation, distress increases when the mother feels she is “serving” two dependants rather than raising one.

In contrast, for younger mothers, the data revealed that a greater distance between the private self and the husband was associated with higher depression. This suggests that for this generation, the crucial factor is not whether the husband acts like a child, but whether he is emotionally aligned with the mother herself. The husband is construed not merely as a family member, but as a key partner in the private sphere. When this emotional or supportive connection is perceived as distant, younger mothers—who often lack the extended family support of the older generation—may feel the weight of intensive childrearing as a solitary burden.

### Research limitations

This study has several limitations. First, the small sample size and the recruitment of participants primarily from affluent urban areas limit generalisability. The generational boundary was also not absolute; some younger mothers born near the cutoff may share similar backgrounds with older mothers. In addition, the absence of a control group of women without children prevents determining whether the observed associations—particularly between financial factors and distress—are specifically related to the experience of motherhood or reflect broader generational patterns among women.

Second, limitations exist regarding the measurement of economic resources. The study operationalised financial status using total household income without assessing women’s individual economic independence. Given the interpretations offered regarding autonomy and neoliberalism, this limits the ability to disentangle family resources from personal economic power. Furthermore, specific financial data—such as educational spending or unpaid family support—were not measured. Consequently, the links between income and mental health remain interpretative. Future research should include more detailed measures of income distribution and objective expenditure measures to clarify these mechanisms.

Third, the cross-sectional design prevents causal conclusions. Regarding measurement, self-report biases apply primarily to the questionnaires; in contrast, the repertory grid technique was specifically used to mitigate such bias by revealing implicit cognitive structures. However, the absence of a “father” element may overemphasise the mother’s role. Finally, given the number of statistical tests, there is a risk of Type I error, necessitating verification in larger, diverse samples.

## Conclusion and future research

This study began with the question of how generational differences in Taiwanese mothers’ construing relate to caregiving, and how such construing may be linked to somatisation and depression. Drawing on Personal Construct Psychology and informed by Taiwan’s generational shifts in maternal norms, the study tested four key hypotheses. While no significant generational differences were found in overall levels of depression or somatisation, or in demographic variables (except children’s age), the findings reveal that the key differences lie in how motherhood, money and relationships are construed.

The results highlight the generational differences. For older mothers, the public self is central. When the public self is too close to the private self or the ideal mother, depression and somatisation increase. This suggests that older mothers feel they must maintain a “good mother” image even in private, which creates emotional strain. Regarding relationships, older mothers appear to integrate extended family into their sense of self: the mother-in-law was not associated with distress, likely reflecting a traditional kinship structure where in-laws are viewed as part of the family rather than external threats. However, tension with their own mothers was significantly linked to somatisation, suggesting that for this generation, unresolved intergenerational conflicts are often expressed through the body rather than verbally articulated.

In contrast, for younger mothers, the private self, autonomy, and high internal standards are central. Their distress reflects a specific dilemma of modern parenting: they have strong, articulate convictions about how to raise their children (reflected in significantly higher tightness), yet they struggle with the cost of this commitment.

Their distress arises from two main sources. First, they suffer from self-discrepancy—when their private experience with the child fails to meet their internal standards, differs from their past identity, or diverges from their own mother’s way of parenting.

Second, they face a tension regarding the centrality of the child. The results showed that younger mothers construed their child as significantly closer to their ideal self, suggesting that the child is central to their ideal identity. However, this deep involvement brings a fear of losing themselves. The data show that when the ideal self becomes too similar to the ideal mother, depression increases. This suggests that while younger mothers are dedicated to intensive parenting, they may be simultaneously anxious about becoming “just a mother” and losing their individual autonomy. Finally, household monthly income was significantly correlated with mental health only for younger mothers, suggesting that for this group, financial resources function less as a mere objective asset and more as a subjective condition for coping with these high, autonomous parenting standards.

These patterns suggest different needs for support. For older mothers, therapy could focus on relaxing the rigid expectations of the public self and validating their own needs. For younger mothers, interventions should address their internal standards and financial anxieties.

Furthermore, these findings offer important insights for government policy on the declining birth rate. The study suggests that simply giving more financial subsidies may not be enough. Since younger mothers’ financial anxiety is often subjective and linked to high standards of intensive mothering, cash alone may never feel like “enough.” Instead, resources should be invested on the “cutting edge”—specifically in bolstering social relationships. Given that younger mothers reported lower social functioning, policy should focus on building supportive social networks to reduce their isolation. By shifting the focus from just giving money to rebuilding a supportive social environment, policy may better address the psychological reasons why women hesitate to have children.

Future research should build on these findings by refining the understanding of economic and relational stressors. First, regarding economic factors, it is crucial to distinguish between objective expenditures—separating educational investments from childcare costs—and subjective financial strain. This would clarify whether money often functions as a proxy for social isolation or a lack of family aid.

Second, the divergence between somatic and depressive symptoms warrants deeper investigation. In the present study, distinct patterns emerged: some element distances were associated exclusively with depression, others with both symptoms, and certain specific distances—such as those involving the mother-in-law for younger mothers or public-private consistency for older mothers—were linked primarily to somatisation. As these differential associations were not the primary focus of the current discussion, future research could explicitly investigate why certain forms of psychological distance or relational tension seem to be preferentially expressed through the body. Are there specific patterns regarding which constructs trigger somatic responses versus depressive ones?

Third, the role of male figures warrants critical attention. The current study focused exclusively on mothers and their female kin, which may introduce a gendered bias by examining maternal distress in isolation from the wider family structure. The absence of fathers and fathers-in-law in the analysis means that the influence of the husband’s role and patriarchal authority was not fully captured. Future research should explicitly include these male figures to avoid reinforcing the assumption that parenting is solely a maternal burden, and to better understand how spousal dynamics and fathers and father-in-law’s presence contribute to—or alleviate—the tensions of motherhood.

Finally, to address the current study’s limitations regarding sample size and urban bias, larger and more diverse samples are needed. Such research would provide critical, dynamic insights for policymakers and clinicians to support the mental well-being of a generation navigating the demands of neoliberal intensive mothering.

## Data Availability

The original contributions presented in the study are included in the article/supplementary material, further inquiries can be directed to the corresponding author.
